# Information Communication Technology as Instrumental Activities of Daily Living for Aging-in-Place in Chinese Older Adults With and Without Cognitive Impairment: The Validation Study of Advanced Instrumental Activities of Daily Living Scale

**DOI:** 10.3389/fneur.2022.746640

**Published:** 2022-03-09

**Authors:** Frank Ho-yin Lai, Angela Yuk-chung Tong, Ada Wai-tung Fung, Kathy Ka-ying Yu, Sharon Sui-lam Wong, Cynthia Yuen-yi Lai, David Wai-kwong Man

**Affiliations:** ^1^Faculty of Health and Life Sciences, Northumbria University, Newcastle upon Tyne, United Kingdom; ^2^Occupational Therapy Department, West Kowloon General Out-Patient Clinic, Hong Kong, Hong Kong SAR, China; ^3^Department of Applied Social Sciences, Faculty of Health and Social Sciences, Hong Kong Polytechnic University, Kowloon, Hong Kong SAR, China; ^4^Salvation Army Hong Kong and Macau Command, Tai Po Multi-Service Centre for Senior Citizen, Hong Kong, Hong Kong SAR, China; ^5^Department of Rehabilitation Sciences, Faculty of Health and Social Sciences, Hong Kong Polytechnic University, Kowloon, Hong Kong SAR, China

**Keywords:** information communication technology, instrumental activities of daily living, aging-in-place, older adults, telemedicine

## Abstract

**Background:**

The capability in applying information communication technology (ICT) is crucial to the functional independence of older peoples of community living nowadays. The proper assessment of individuals' capability of ICT application is the corner stone for the future development of telemedicine in our aging population.

**Methods:**

With the recruitment of 300 participants of different functional and social background in home-living, hostel-living, and care-and-attention home living; and through assessing the ability of individuals in instrumental activities of daily living and cognitive assessments, this study aimed at capturing the content validity and construct validity of the Advanced Instrumental Activities of Daily Living (AIADL scale). In addition, this study assess the ability of older peoples in applying ICT and how the functional and social background affects their independence in aging-in-place.

**Results:**

The AIADL scale showed good test-retest reliability and good-to-excellent internal consistency. To determine if items of the AIADL scale measure various aspects of community living, exploratory factor analysis revealed a two-factor structure with “home living and management” and “community living”. Validity analysis with the known-groups method showed a high overall accuracy of prediction of individuals' capability of independent living in the community.

**Conclusions:**

The AIADL scale is a valid and reliable instrument to assess the ability of older adults in handling ICT as part of their instrumental activities in daily living. The scale can reflect capability of older peoples in applying ICT. This instrument can serve as a reference in measuring readiness of individuals in receiving telemedicine and their ability of aging-in-place.

## Background

Activities of daily living (ADL) describe basic but essential everyday activities of self-care, such as bathing, dressing, and feeding (1). Instrumental activities of daily living (IADL) describe activities necessary for adaptation to the environment and emphasize community activities, such as shopping, cooking, transportation, and other types of activities including housekeeping. These activities are key life tasks that older adults need to manage to live in the community and be functionally independent (2, 3). The activities of IADL are more cognitively influenced (4) and are important parameters for successful aging in place of older adults (5, 6). Advanced activities of daily living (AADL), on the other hand, represent activities that involve superior cognitive abilities along with adequate physical and social functioning that could enable an individual to maintain his or her own self-identity through the development of various social roles, such as event-planning and participation within the community (7). Occupational therapists play an important role in assisting older adults to overcome functional decline of individuals and support engagement of their own life roles in the community (8). Reflecting on the fact that information communication technology (ICT) is becoming an increasingly inseparable part of our modern lives, we have further coined the term advanced instrumental activities of daily living (AIADL) as IADLs that have taken into account the technological competencies necessary for independent living within today's community.

The Lawton Instrumental Activities of Daily Living (Lawton IADL) scale is a well-known and classical instrument in assessing the independent living skills of individuals (9–11). Due to its easier apprehensible and less time demanding in administration (11, 12), the Lawton IADL still outweighs more recently developed IADL measures, such as the Assessment of Living Skills and Resource (ALSAR) (13, 14). The Lawton IADL has been cited by over 3,000 published studies (11) and has considerable evidence of its reliability and concurrent validity (15, 16). Its Chinese version, namely the Lawton Instrumental Activities of Daily Living—Chinese Version (IADL-CV) was validated in 2002 using data from 155 older adults living in homes for the aged and care-and-attention homes (16). The IADL-CV consists of nine items: use of telephone, transportation, shopping, medication management, money management, meal preparation, housework, laundry, and handyman work. It was shown to be a reliable instrument for assessing the ability of older adults to live independently in the community. With the use of the known-groups method, the IADL-CV had been validated with a one-factor structure (16). However, the psychometric properties of IADL-CV have not been further examined in the past decades and it does not measure ability of individuals in applying ICT (13).

Applied technology, such as the use of ICT and smartphone applications, has had a huge impact on the world and on lifestyles of individuals. Ability in handling these technologies is not only considered essential for daily functioning but also plays a role in formulating an individual's sense of independence in the community, thus increasing the quality of life of an individual (17, 18). This ability is getting more and more common in the contemporary digitalized world (19, 20) and regarded as a core essential skill for the older people (21–24) and has been regarded as the corner stone for the development of telemedicine in dementia care and treatment (21, 22). Due to the huge gap of existing daily living measurement tools, such as the Lawton IADL and the IADL-CV, which had not been designed to cater for the currently technologically heavy times (13, 15, 25), we should therefore have an instrument in place that can evaluate functions of individuals in the contemporary community nowadays.

This study aimed at assessing the ability of older adults' ICT application, and how their functional and social background affects the independence of individuals in aging-in-place and to capture the content validity and construct validity of the Advanced Instrumental Activities of Daily Living (AIADL scale). Instead of sacrificing the already established psychometric properties of the IADL-CV that had developed its framework from the well-known Lawton IADL, this study enriched the content of the IADL-CV by adding to it the relevant items involving ICT and smartphone applications for engaging in IADLs nowadays, creating an updated instrument called the AIADL scale that can capture the technological aspect of everyday living of the Chinese older people. In validating the AIADL, the classical test theory was adopted (26, 27) to examine and test (1) the degree of clarity, understandability, and relevance (i.e., content validity), (2) the test-retest reliability score of the AIADL scale, (3) the degree of the inter-relatedness among the AIADL scale items, such as internal consistency, (4) the factor structure of the AIADL scale by exploratory factor analysis, (5) the correlation between the Lawton IADL score and the AIADL scale measure, and (6) to determine the construct validity and by using the AIADL scale to predict the residence of older adults with known-groups method [in parallel with the method adopted by the validation of IADL-CV (16)].

## Methods

### Participant Selection and Ethics Consideration

To ensure generalizability of the research findings that apply to older people in the community, participants were recruited from members of several day activity centers located in six different districts in Hong Kong. These participants included home-living participants (HL) who are functionally and socially independent community dwellers living in their own homes, hostel-living participants (HE) who are independent in terms of self-care and community living but with a need for social support, and care-and-attention home living participants (C&A) who need environmental support as well as assistance with their daily functioning. A total of 100 participants were recruited through purposeful sampling from a local non-government organization through an advertisement and all of them had to complete the MoCA-HK, the CDAD, Lawton IADL, and the AIADL scale questionnaires. In examining test-retest reliability of the AIADL scale, the HL group was asked to fill out the AIADL scale again 1 week later. In analyzing the factor structure, performances of both the HL and the HE group on the tests were used so as to conduct an exploratory factor analysis. Finally, in exploring the group differences among the three groups, known-group analysis was employed to compare the performances of groups on the MoCA-HK, the CDAD, the Lawton IADL, and the AIADL scale.

Prior to their participation in this study, written consents were sought from every participant with their first-degree relatives as witnesses. The inclusion criteria were: (a) ages between 65 and 80 years inclusive, which covered more than 80% of the older people in Hong Kong, (b) the ability to understand verbal and written Chinese instructions, and (c) the ability and willingness to provide written consent and sign the relevant document. The following exclusion criteria were applied: (a) participants with a history of substance abuse, such as alcohol, drugs, or any medication/substances indicative of chronic abuse, so as to prevent any possible craving behaviors from occurring that could lead to biased results; (b) participants with major neurological disorders, such as stroke and head injury, which could have a more direct impact on their IADL performances. Approval was given by the university research ethics committee and the study was conducted according to the Declaration of Helsinki.

### Measurements

The items of the AIADL scale were initially developed by a panel of experts. The panel then reviewed the items content and cultural relevance of these items and explore how these were relevant to aging. The experts in the panel, who have experience of more than 20 years in the frontline domiciliary healthcare services, examined each item in the IADL-CV. The panel used the tailor-made questionnaire to evaluate the relevance of the IADL-CV to IADL for community-living older adults. The relevance of each item was assessed by a self-reported questionnaire using a visual analog scale ranging from 1 to 10 (1 = no relevance; 10 = cultural relevance). The panel agreed that a score of <7 indicated that an item may not be relevant. Panel members recommended that the item on the adoption and handling of ICT should be added to measure ability of individuals in community living. Additionally, the concept of using stored value smart card for electronic payments should be added for money management. With the modification of the IADL-CV items, the AIADL scale is a 10-item instrument to be used in assessing the IADLs of older people in the community. These ten items include use of information communication technology (in accessing the internet to obtain information), use of landline telephone, transportation, shopping, medication management, using electronic payments and money management, meal preparation, housework, laundry, and handyman work. The score ratings are from 0 – dependent, 1 – with help to 2 – independent, accumulatively ranging from 0 to 20. Higher scores indicate higher levels of functional independence in performing IADLs. Additionally, the cognitive function of individuals was screened using the Hong Kong Montreal Cognitive Assessment (HK-MoCA) (28), scoring 19 or less would be classified as having cognitive impairment. Furthermore, the physical disability and executive function of individuals were assessed by the Chinese version of the Disability Assessment for Dementia (CDAD) (29), their ability in instrumental activities of daily living Lawton IADL score (9). These were used to assess the convergent validity of the AIADL scale, as the both CDAD and Lawton IADL has been well recognized for the “golden” measurement of individuals' instrumental activities of daily living (29).

### Statistical Plan

**Figure 1:**
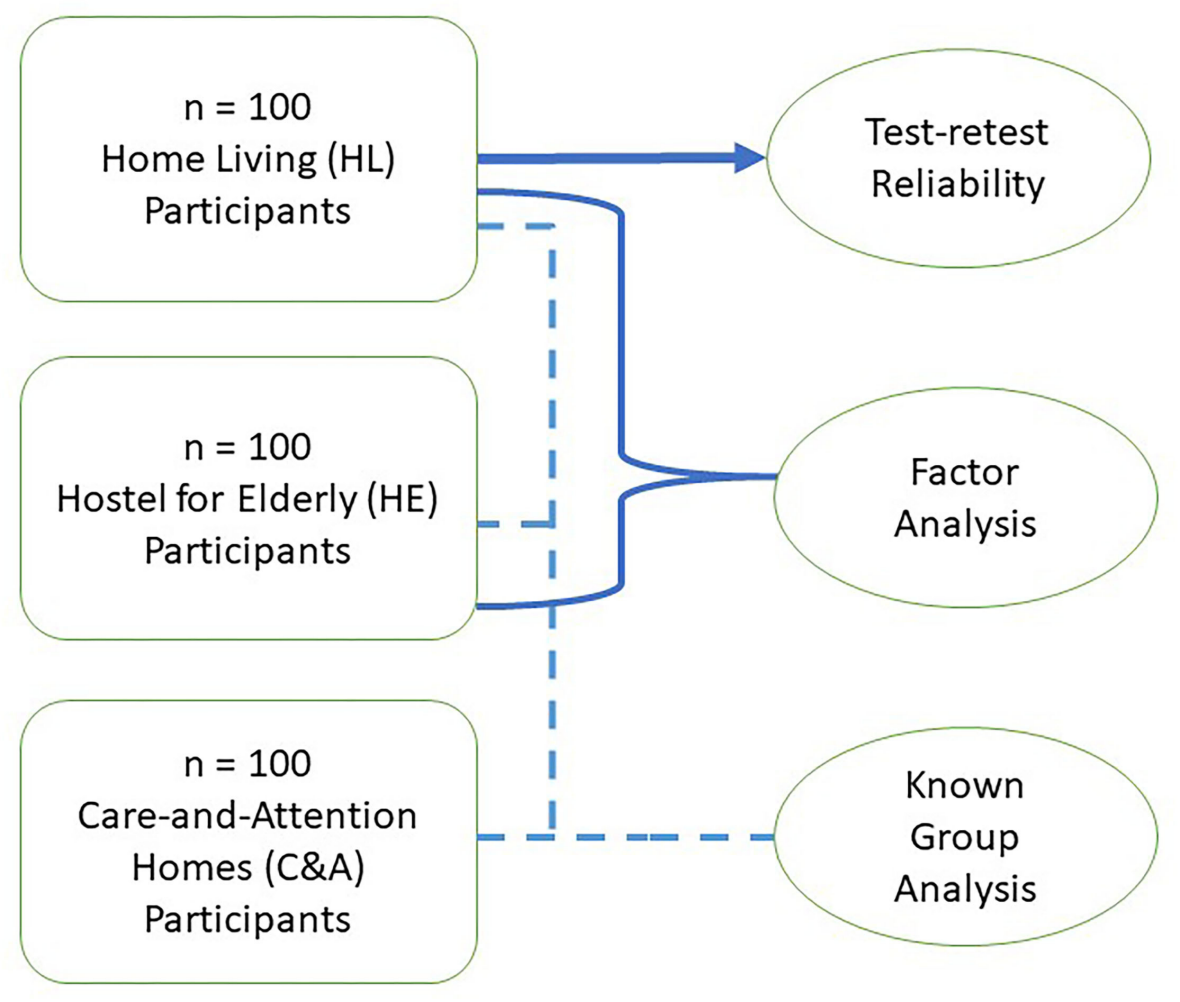
Flow diagram to demonstrate recruitment of participants.

The AIADL scale was tested for its degrees of clarity, understandability, and relevance. Moreover, kappa coefficient was used in interpreting the degree of agreement of expert panels for these items on the AIADL scale. The content validity and cultural relevance of the AIADL scale were measured by the content validity index. In construct validation, there are three different groups of participants. HL participants who are independent dwellers living in their own homes. HE participants who are independent in terms of self-care and community living but with social support needs. These two groups of participants provided their self-ratings on the AIADL scale. C&A dwellers need environmental support and assistance in IADL tasks. HL and HE participants self-completed the AIADL scale while C&A participants were helped by the interviewer in completing the AIADL scale. The recruitment of participants is as shown in the flow diagram in Figure 1.

Demographic information, socio-demographic, and health history were collected through the membership registration of the NGO. Data analyses were conducted using IBM-SPSS (version 23) on Windows 10 operating system (OS). Standard descriptive statistics were computed for continuous data and frequency distributions for non-continuous data. The Shapiro–Wilk test was used to check whether or not a continuous variable follows a normal distribution. Parametric analysis would be employed as far as possible, with data transformation to better comply with mathematical assumptions of parametric analysis whenever appropriate. Statistical significance of 0.05 would be applied throughout. The intra-class correlation coefficient (ICC) would measure the reliability and internal consistency, by two-way mixed-effects model and absolute-agreement model, of ratings on the AIADL scale. We used chi-square tests to compare the frequency distributions between the different groups of participants. For construct comparison, Pearson's correlation coefficients were computed to compare scores on cognitive (HK-MoCA) and functional disability and executive function (CDAD) and instrumental activities of daily living (Lawton-IADL) parameters. To compare if there was any difference among the three groups, ANOVA comparison and *post-hoc* analysis of cognitive function and the AIADL scale mean scores among these groups were conducted. Moreover, in determining if items of the AIADL scale measure various aspects of community living of the participants who are independent in respect of community living, the exploratory factor analysis using a principal-axis factor extraction was conducted to determine the factor structure of the AIADL scale. Bartlett's test of sphericity was used to test if the correlation matrix was an identity matrix, which indicated variables are unrelated and therefore unsuitable for structure detection. Statistical significance of 0.05 indicated that factor analysis could be useful. The Kaiser-Meyer-Olkin (KMO) test was used to determine the sampling adequacy of the data that were used for factor analysis. Validity analysis by the known-groups method was used to predict the accuracy of their residence in the community (16).

### Sample Size Estimation

Power analysis was performed using G^*^Power based on the previous reference study of the IADL-CV (16) and calculated with a medium effect size = 0.4, statistical significance = 5%, and estimated power = 0.8. G^*^Power indicates that the required sample size per group is 84 for test-retest reliability in older people who are independent in community-living. Each participant was asked to complete the AIADL scale two times, the interval between the two measures was 1 week for test-retest reliability. For factor analysis of independent community-living older people, the present exploratory factor analysis used in determining the factor structure of the AIADL scale fulfilled the “old school” theory on the number of cases per variable (N/p), with recommended ratios ranging from 3:1–6:1 (30) to 20:1 (31). In particular, Hair and colleagues had advised researchers to obtain the highest cases-per-variable ratio possible to minimize the chance of overfitting the data. Therefore, ninety participants per group for HL and HE were required in producing small to medium effect size = 0.3. Taking 10% attrition rate, 100 HL and 100 HE participants were recruited. Another 100 functionally dependent participants from C&A were recruited for validity analysis by predicting the social group through using the known-groups method.

## Results

### Content Validity of the AIADL Scale

To assess content validity of the AIADL scale, 10 Chinese-speaking HL older people (6 women and 4 men; ages ranged from 65 to 75 years, mean = 68.76 years, SD = 2.76) were recruited from a day activity center to complete a survey. The education of participants ranged from primary to tertiary levels with the mean of the participants' education levels with 8.32 years. The panel of experts consisted of five members (three occupational therapists and two community nurses) with experience of more than 20 years (with mean 20.23 years) in domiciliary healthcare and they actually used these tools in their line of work. After revealing the feedback in the survey, panel members discussed all items of the AIADL One item on adoption and handling of ICT was added to the AIADL scale. Additionally, the concept of using stored value smart card for electronic payments was refined under the category of money management, so as to increase the comprehensibility and relevance to money management nowadays. All ten items showed satisfactory clarity and understandability of presentation in the AIADL (with a mean score > 7 out of 10) except the item on handyman work which scored 6.82 out of 10 (SD = 0.29). This was referred back to the panel for further discussion and confirmed to be retained.

For the panel survey, the kappa score was used to indicate the level of agreement of item and content of AIADL between the panel members. As depicted in Table 1, all items of the AIADL scale result with the Kappa score range from 0.61 to 0.75, which indicate that there are moderate to substantial agreement of items among the AIADL scale (32). Moreover, all of the item-level content validity index (I-CVI) indicated the item-level content validity ≥ 0.81, and the scale-level content validity index based on the average method (S-CVI/Ave) = 0.83 indicated good content validity and cultural relevance of the new AIADL scale.

**Table 1:** Agreement of items by expert panel (*n* = 10).

**Item of AIADL**	**Kappa score (95% confidence interval)**	**I-CVI (item-level content validity index)**
1. Use of telephone	0.75 (0.43–0.95)	0.83
2. Use of information communication technology (*new item)	0.74 (0.42–0.94)	0.81
3. Transportation	0.72 (0.45–0.89)	0.86
4. Shopping	0.68 (0.46–0.92)	0.81
5. Meal preparation	0.66 (0.47–0.93)	0.86
6. Housework	0.71 (0.46–0.92)	0.81
7. Handyman work	0.65 (0.43–0.95)	0.84
8. Laundry	0.67 (0.38–0.86)	0.84
9. Medication management	0.62 (0.41–0.87)	0.82
10. Money management (^#^refined item)	0.62 (0.43–0.82)	0.83

### Test-Retest Reliability of the AIADL Scale Assessment

The characteristics of these three groups are depicted in Table 2. Since we had a relatively large sample size and a Shapiro–Wilk test was performed and did not show evidence of non-normality (W = 0.92, *p* = 0.11; W = 0.79, *p* = 0.35; W = 0.86, *p* = 0.55 in HL, HE, and C&A, respectively). Based on this outcome, and after visual examination of the histogram of the QQ plot, we decided to use a parametric test.

**Table 2:** Characteristics of recruited subjects.

	**Female (*n*)**	**Male** **(*n*)**	**Age range** **(mean ±SD)**	**MoCA (mean ±SD)**	**CDAD (mean ±SD)**	**Lawton IADL (mean ±SD)**	**AIADL (mean ±SD)**
Home-living participants (HL)	65	35	65–75 (69.71 ± 2.58)	23.89 ± 1.65	0.92 ± 0.03	16.34 ± 0.23	19.52 ± 1.26
Hostel-living participants (HE)	53	47	67–77 (68.34 ± 1.47)	23.72 ± 1.39	0.91 ± 0.03	15.78 ± 0.32	19.48 ± 1.21
Care-and-attention home living participants (C&A)	45	55	66–80 (71.23 ± 7.38)	14.29 ± 2.19	0.42 ± 0.09	11.21 ± 0.23	13.28 ± 2.84

In test-retest reliability analysis, 100 participants from the HL group (65 women and 35 men; ages ranged from 65 to 75 years, mean = 69.71 years, SD = 2.58) were recruited. They had a MoCA score of mean = 23.89, SD =1.65, the CDAD score of mean = 0.92, SD = 0.03. The Lawton IADL score of mean = 16.34, SD = 0.23. These scores indicated these group of participants were having intact cognitive functions. The AIADL scale was repeated 1 week after the pre-test by the 100 participants. The ICC and 95% *CI*s were calculated on the basis of two-way mixed-effects model. There was good test-retest reliability with an ICC of 0.88 from the AIADL scale summation score (individual item ICCs ranging from 0.86 to 0.92, and 95% *CI*: 0.84–0.95) as shown in Table 3. There was good to excellent internal consistency (Cronbach's alpha = 0.94).

**Table 3:** Reliability testing of the AIADL scale (*n* = 100, home living participants).

**Items of AIADL**	**Test-retest reliability (ICC) (*n* = 100)**
1. Use of telephone	0.90 (95% C.I. = 0.89–0.91)
2. Use of Information communication technology (*new item)	0.86 (95% C.I. = 0.84–0.91)
3. Transportation	0.91 (95% C.I. = 0.86–0.94)
4. Shopping	0.91 (95% C.I. = 0.85–0.95)
5. Meal preparation	0.90 (95% C.I. = 0.86–0.91)
6. Housework	0.92 (95% C.I. = 0.87–0.93)
7. Handyman work	0.90 (95% C.I. = 0.88–0.91)
8. Laundry	0.92 (95% C.I. = 0.89–0.93)
9. Medication management	0.89 (95% C.I. = 0.84–0.91)
10. Money management (^#^refined item)	0.88 (95% C.I. = 0.86–0.93)

### Factor Analysis of the AIADL Scale

In analyzing the factor structure, apart from the 100 HL independent community-dwelling participants that were recruited initially, another 100 independent community living HE participants (53 women and 47 men; ages ranged from 67 to 77, mean = 68.34, SD = 1.47; with MoCA score with mean = 23.72, SD = 1.39, CDAD score with mean = 0.91, SD = 0.03, and the Lawton IADL score of mean = 15.78, SD = 0.32) were recruited. The mean score of the AIADL scale of the HL group was 19.52 (SD = 1.26) and the HE group was 19.48 (SD = 1.21). There was good linear relationship between individual items on the AIADL scale (Pearson's *r* ranging from 0.72 to 0.91), item-factor correlation with Pearson's *r* ranged from 0.78 to 0.90, and item-total correlation ranged from 0.79 to 0.89. To test for the correlation matrix of variables, Bartlett's test of sphericity was used to establish the adequacy of the dataset. All items on the AIADL scale showed a *p* of < 0.05. KMO measure of sample adequacy showed with 0.82, which indicated a factor analysis that would be useful with the collected data.

Category quantification was applied to treat the levels of the trichotomized data directly as values from a continuous distribution. The exploratory factor analysis using a principal-axis factor extraction was conducted to determine the factor structure. Direct oblimin rotation methods were used and created two factors with sums of squared loadings ranging from 0.72 to 0.81. Two dimensions were yielded from the factor analysis, their loading is depicted in Table 4. The first dimension had an Eigen value of 3.95 (with 95% *CI* from 2.47 to 4.21) which contributed 45.60% of the variance; the second dimension had an Eigen value of 1.98 (with 95% *CI* from 1.21 to 3.21), which contributed 39.92% of the variance.

**Table 4:** Factor loading of the AIADL scale [*n* = 100, home living (HL) and *n* = 100, hostel for older people (HE)].

**Items of AIADL**			**Item scores (HL)**	**Item scores (HE)**	**Factor 1**	**Factor 2**
1. Use of telephone			1.81 ± 0.12	1.80 ± 0.11	0.72	0.11
2. Use of information communication technology (*new item)			1.67 ± 0.23	1.65 ± 0.18	0.17	0.72
3. Transportation			1.67 ± 0.23	1.66 ± 0.21	0.12	0.72
4. Shopping			1.63 ± 0.23	1.63 ± 0.21	0.12	0.72
5. Meal preparation			1.72 ± 0.09	1.72 ± 0.11	0.81	0.07
6. Housework			1.34 ± 0.42	1.34 ± 0.41	0.72	0.12
7. Handyman work			1.47 ± 0.39	1.47 ±0.38	0.72	0.21
8. Laundry			1.62 ± 0.23	1.63 ± 0.32	0.72	0.09
9. Medication management			1.67 ± 0.21	1.65 ± 0.22	0.77	0.12
10. Money management (^#^refined item)			1.72 ± 0.23	1.71 ± 0.23	0.11	0.72
Total score			19.52 ± 1.26	19.48 ± 1.21		
**Confidence intervals (CIs) for eigenvalues**						
**Factor number**	**Observed eigenvalue**	**95% CI**				
1	3.95	(2.47–4.21)				
2	1.98	(1.21–3.21)				

Upon thorough discussions among the expert panel and the research team, factor one was labeled “home living and management, ” which represented IADL tasks that are typically performed within the household, and included six items: use of telephone, meal preparation, housework, handyman work, laundry, and medication management. Factor two was named as “community living, ” which represented other IADL tasks that are generally done within the community outside the household, and consisted of four items: transportation, shopping, money management, and use of mobile electronic communication devices. The ranges of item total correlation were from 0.75 to 0.82 (for “home living and management”), and 0.71 to 0.83 (for “community living”). In measuring the internal consistency of these two individual factors and the overall AIADL scale, the Cronbach's alphas were 0.96, 0.94, and 0.94, respectively. The high internal consistency suggests that the two factors and the overall AIADL scale measure the same construct. Moreover, the Lawton IADL showed higher correlation with the AIADL scale (*r* = 0.87, *p* < 0.01), with “Home living and management” factor (*r* = 0.89, *p* < 0.01), and “Community Living” factor (*r* = 0.73, *p* < 0.01). The distribution of items' score is depicted in Table 4.

### Examine Group Difference From Three Types of Residences

In examining if there were group differences, apart from the HL and HE participants, we recruited another 100 C&A participants, 45 women and 55 men, with ages ranging from 66 to 80 years (mean = 71.23, SD = 7.38); MoCA score with mean = 14.29, SD = 2.19, CDAD score with mean = 0.42, SD = 0.09 and the Lawton IADL score of mean = 11.21 ± 0.23. Their AIADL scale score was 13.28 (SD = 2.84).

In accordance with the methodology in validating the IADL-CV (16), by using the known-groups method, the AIADL scale was used to predict participants into their corresponding living institutions, i.e., HL, HE, and C&A homes. Table 5 shows a high accuracy of older adults' residence in the community (91.67%). This figure came from concordant pairs (92 + 88 + 95)/300. The correlation coefficient between the AIADL scale scores and known group was 0.85, a correlation matrix was constructed using the cognitive functions of participants and factors of the AIADL scale. Cognitive function showed a significant correlation with home living and management (*r* = 0.78, *p* < 0.001), and with community living (*r* = 0.72, *p* < 0.01).

**Table 5:** Classification results of grouping [with *N* = 300; with home living (HL): *n* = 100, hostel for older people (HE): *n* = 100, care and attention home (C&A): *n* =100].

			Predicted group membership^*^		**C&A**	
		**Institution**	**HL**	**HE**		***n***
Original	Count	HL	92	6	2	100
		HE	10	88	2	100
		C&A	1	4	95	100
	%	HL	92.0%	6.0%	2.0%	100%
		HE	10.0%	88.0%	100.0	100%
		C&A	1.0%	4.0%	95.0%	100%

*91.67% [Concordant pairs = (92 + 88 + 95)/300] of original grouped cases correctly classified*.

* *In cross validation, each case is classified by the functions derived from all cases other than that case*.

A one-way ANOVA among the subjects was conducted to compare the effect of groups on AIADL and cognitive conditions. There was a significant effect of grouping on AIADL at the *p* < 0.05 level for the three groups [*F*_(2,297)_ = 202, *p* = 0.03]. A *post-hoc* comparison using the Tukey's honesty significant difference (HSD) test indicated that the mean score for the C&A group (M =13.28, SD = 2.84) was significantly different from the HL and HE group (M = 19.52, SD = 1.26 and M = 19.48, SD = 1.21, respectively). Similarly, there was a significant effect of grouping on cognitive function at the *p* < 0.05 level for the three groups [*F*_(2,297)_ = 189, *p* = 0.03]. A *post-hoc* comparison using the Tukey's HSD test indicated that the mean score for the C&A group (M =14.29, SD = 2.19) was significantly different from the HL and HE group (M = 23.89, SD = 1.65 and M = 23.72, SD = 1.39, respectively). However, there was no significant difference in both AIADL and cognitive functions from HL and HE participants.

In convergent validity, the score of the AIADL scale had a high correlation with the cognitive construct- the MoCA-HK (*r* = 0.86, *p* = 0.02), and the functional construct -the CDAD (*r* = 0.85, *p* = 0.01); the Lawton IADL (*r* = 0.96, *p* = 0.01). The AIADL scale was shown to be reliable and valid in assessing the daily function of community-residing older adults.

## Discussion

Aging in place is a process that involves both the person and the environment; it is a continuous dynamic interaction as both the person and the environment changes. With the influence of ICT, our living environment has changed substantially (20, 25). Rehabilitation practitioners should be sensitive to the changing environment, cultural, and social factors over time. ICT, such as smartphone application or other mobile electronic devices (33), use of stored value smart cards for making electronic payments (34), and Internet browsing are considered essential for older adults in the community nowadays (15). Nevertheless, this trend of daily community living with technology has been constantly developing in “young old” population (35). However, the conventional assessment on IADLs, such as the Lawton IADL cannot totally reflect such trends.

In aging theory, capabilities and limitations of people change across their lifespan. There are general patterns of physical and cognitive changes that occur with age. However, the decline of cognitive functions may not be easily noticeable until later stages of neuro-cognitive disorders. This study evaluated individuals aged 65–80 of their abilities with cognitive functions in performing contemporary IADLs and illustrated the importance of both cognitive functions and physical functions in the execution of IADLs for older adults. The selected age range effectively represented the majority of the older people in Hong Kong. Specifically, the minimum bound was set at 65 years of age as it is currently regarded as the defining age of the older people in Hong Kong (36), which is also the minimum age of acceptance into either HE or C&A for those in need (37). It is also the age at which general incidence of dementia occur, which is known to have a significant association with decline in functional status (38, 39). On the other hand, the upper limit of 80 years old was set in accordance with the general life expectancies in Hong Kong, which were 82.2 years for male and 88.1 years for female as of 2019 (40). In ensuring that the recruited subjects were well-suited for the purpose of this study, prospective candidates who were chronic substance abusers or who had major neurological dysfunctions were excluded from our selection, since their cognitive functions and abilities in performing IADLs may significantly deviate from the norm and subsequently lead to unjustified results. In particular, chronic abusers, especially those diagnosed with substance use disorders, are characterized by their inabilities to meet personal or occupational obligations, and they may also withdraw from social activities or even cause ongoing legal problems, such as thievery as a consequence of their drug use. All of these would compromise their abilities in engaging within the community in an orderly manner as well as affect their abilities in performing certain IADLs properly. Therefore, they had all been excluded from participating in this study.

To develop the AIADL scale and establish it as a new measure of older adults' IADL abilities in the digital age of today, we have first recruited experts with over 20 years of clinical experiences to help assess the content validity and cultural relevance of each of the items on the IADL-CV. As the IADL-CV was validated back in 2002, the rich clinical experiences gained by these experts in the past 20 years where they had adopted it as a means of functional ability assessment would ensure that they are the best qualified professionals to provide an expert opinion on the validity of its individual items, and are therefore sufficiently capable of evaluating and scrutinizing the choice of items on the AIADL scale. Furthermore, being practitioners themselves, these experts are well-adapted to the evolvement and utilization of new technologies within the healthcare settings. This has enabled them to develop a keen sense of identifying the types of technologies that are particularly accessible to the older people in their everyday living, which is a highly desirable skill that was constantly employed in the process of devising the AIADL scale. Having established agreement among panel members in finalizing the AIADL scale, which consisted of 10 items with each attaining a kappa score in the range of 0.62–0.75, as well as validating its content validity to be used within the context of Hong Kong, the AIADL scale was further tested for its reliability and validity by our recruited participants. The AIADL scale showed comparable standards of disability and cognitive measures to other well-cited literature (41, 42), and over-weighting the conventional IADL measure (41, 43). Our findings echoed previous literature which documented that IADLs demand performance in cognitive domains, such as memory, attention, and executive function (44).

To maintain coherence with the research design of the IADL-CV (16), HE participants were recruited in the current study. Moreover, the present validation study of the AIADL scale transcended the IADL-CV by recruiting a significantly larger group of participants and wider population spectrum (total participant population = 300) that can provide a more laudable evidence in aging research. The coverage of participants nearly encompassed the main groups of older adults in our community. Taking into consideration this wide spectrum of coverage, the capability to use mobile technology in handling communication and finance was considered as an important ability that is much needed by the older adults for them to live independently in the community (41).

In respect of test-retest reliability, using data from the HL group who had been asked to complete the AIADL scale at two different time points separated by a week apart, the AIADL was found to have reached good test-retest reliability with an ICC of 0.88, and good to excellent internal consistency with a Cronbach's alpha of 0.94. These results were comparable to those presented by Tong and Man (16) in their validation study of the IADL-CV, which had an ICC of 0.90 and a Cronbach's alpha of 0.86. Our results on reliability and internal consistency of the AIADL scale were well above the standard that (45) had stated.

Similar to the findings of Tong and Man (16) on the IADL-CV, using the known-groups method, a high accuracy of prediction on the residencies of older adults within the community was found when the AIADL scale was adopted. Specifically, the AIADL scale had correctly predicted participants from the HL, HE, and C&A groups into their corresponding residencies 92.0, 88.0, and 95.0% of the times, respectively, reaching an overall accuracy rate of 91.67%. In contrast, although the IADL-CV had a high accuracy of prediction rate for the HE group (94%), the overall accuracy of prediction was not as high (78%).

The scale can differentiate between seniors of differing needs and abilities. This study documented and justified that participants living in different residency types would show different patterns of scoring on the AIADL scale. It is reasonable to believe that participants in the HE and C&A groups should maintain communication with their relatives and friends or even for the purpose of handling emergencies *via* ICT and smartphone application. The present study would help the authors in their future work by identifying tasks and activities that differ among various living contexts. Inability of individuals in performing certain IADLs can be referred to occupational therapists so that they could provide further remediation training and compensatory intervention to them. This can further enhance the capability of individuals to cope with aging in place. Moreover, it is interesting to note the discordant pairs (10 + 1 + 6 + 4 + 2 + 2)/300= 8.33% as shown in Table 5. Ten people who had been classified as HE were predicted as HL. This can be partially explained by the fact that the functional levels of older adults in the HE group were similar to the HL group, except that HE required social support.

Measuring inability of an individual to perform IADLs is important not just in determining the level of assistance required, but as a metric for a variety of services and programs related to caring for the older adults and for those with disabilities. Many Chinese older adults wish to remain living in the community they have occupied for decades, while others have already downsized or moved into institutional care facilities. The validated AIADL scale helps rehabilitation practitioners assess ability of individuals to successfully manage their IADLs in the contemporary community, a key element that supports the current age-in-place plans. To achieve the goal of aging-in-place, it is necessary to plan for the future and be prepared to respond to changes that come with aging. The validated AIADL scale will serve as a useful reference tool to help identify important areas that are of priority for the future planning of our aging populations. Occupational therapists can also assist with the planning process by making recommendations to maximize independence and helping individuals overcome any areas of difficulty. Recommendations may relate to the care plan of individuals, the use of assistive devices, suggesting activities adaptation, or linking to community support services and programs.

## Study Limitation

The limitations of the AIADL scale assessment include the fact that it is based on the self-report method of administration rather than the performance of functional tasks. This may lead to either overestimation or under-estimation of older adults' abilities (46). It is worthy that further study on how AIADL compare with other IADL tools. Moreover, the lack of comparisons for measuring the efficacy of using ICT devices objectively limits the generalizability of the study findings. Furthermore, in the test-retest reliability assessment, the 1-week test-retest interval could be lengthened to 3 weeks to alleviate the memory and learning effect. Further studies can address this gap to further enhance the quality of the AIADL scale's assessment.

## Conclusion

The ability in applying ICT is crucial to functional independence and effective aging-in-place of older peoples. Their adoption and handling of ICT should be a crucial parameter to be addressed. It is believed that proper assessment is the corner stone for the future development of telemedicine in our aging population. Healthcare practitioners should also be sensitive to the changing environment, as well as the cultural and social factors around our aging population over time. The two-factor structure of the AIADL scale assessment, “home living and management” and “community living, ” is shown to be a valid and reliable instrument that can be used to assess the IADL abilities of older adults in this contemporary community.

## Data Availability Statement

The raw data supporting the conclusions of this article will be made available by the authors upon request, without undue reservation.

## Ethics Statement

The studies involving human participants were reviewed and approved by The Hong Kong Polytechnic University. The patients/participants provided their written informed consent to participate in this study.

## Author Contributions

FL coordinated the whole study, developed the research idea, executed the research plan, and monitored the progress. FL, CL, and DM had substantially contributed to the conception and design of the work and analysis and interpretation of research data. AT and AF had conducted the literature review. KY and FL assisted in data collection and including the arrangement for interventions for participants. AT and KY assisted in the literature search and served as the blinded assessors in the study. SW had helped in the earlier drafts of the manuscript and assisted in subsequent revision of the text. They have thus had contributed significant to the article's intellectual content. All authors have agreed to be accountable for all aspects of the work in ensuring that questions related to the accuracy or integrity of any part of the work are appropriately investigated and resolved. All authors contributed to the article and approved the submitted version.

## Funding

This study was supported by Innovation and Technology Fund for Better Living (FBL), under the project VR & AI-based Mobile Apps in Enhancing Independence of Daily Living in Older Adults and People with Early Dementia (Program Code: ITB/FBL/2004/19/P).

## Conflict of Interest

The authors declare that the research was conducted in the absence of any commercial or financial relationships that could be construed as a potential conflict of interest.

## Publisher's Note

All claims expressed in this article are solely those of the authors and do not necessarily represent those of their affiliated organizations, or those of the publisher, the editors and the reviewers. Any product that may be evaluated in this article, or claim that may be made by its manufacturer, is not guaranteed or endorsed by the publisher.

## Abbreviations

AIADL: The Advanced Instrumental Activities of Daily Living
AIADL scale: The Instrument of Advanced Instrumental Activities of Daily Living
IADL: The Instrumental Activities of Daily Living
Lawton IADL: The Lawton Instrumental Activities of Daily Living
ALSAR: The Assessment of Living Skills and Resource
IADL-CV: The Lawton Instrumental Activities of Daily Living—Chinese Version
HK-MoCA: The Hong Kong Montreal Cognitive Assessment
CDAD: The Chinese version of the Disability Assessment for Dementia
HL: Participants who were Home-Living
HE: Participants from Hostels for the Older People
C&A: Participants from Care-and-Attention Homes.
