# Supplementary material for: Information Communication Technology as Instrumental Activities of Daily Living for Aging-in-Place in Chinese Older Adults With and Without Cognitive Impairment: The Validation Study of Advanced Instrumental Activities of Daily Living Scale
Source: Front Neurol. 2022 Mar 9;13:746640. doi: 10.3389/fneur.2022.746640 (PMC8959306; doi:10.3389/fneur.2022.746640)
Supplement: Supplementary file 1 [file Data_Sheet_1.pdf]

The Advanced Instrumental Activities of Daily Living (AIADL) Scale

Item	2<br>Independent	1<br>With Help	0<br>Dependent
**1. Use of telephone**<br>(Use landline telephone to dial and receive phone calls)			
**2. Use of Information Communication Technology**<br>(Use mobile communication devices in accessing information from the internet and for communication with others)			
**3. Transportation**<br>(Use public transport in own living district)			
**4. Shopping**<br>(Perform shopping in grocery store, convenience store or supermarket)			
**5. Meal Preparation**<br>(Plan, organize and prepare meal)			
**6. Housework**<br>(Handle household chores of own dwelling place)			
**7. Handyman Work**<br>(Perform handyman work of own dwelling place)			
**8. Laundry**<br>(Handle laundry tasks with or without use of washing machine)			
**9. Medication Management**<br>(Manage correct dose of medicine with correct regime)			
**10. Money Management**<br>(Use electronic and cash money management and payment)			

Total Score : / out of 20
